# Weber’s Law and the Scalar Property of Timing: A Test of Canine Timing

**DOI:** 10.3390/ani9100801

**Published:** 2019-10-14

**Authors:** Jessica H. Cliff, Surrey M. K. Jackson, James S. McEwan, Lewis A. Bizo

**Affiliations:** 1 School of Psychology, The University of Waikato/*Te Whare Whãnanga o Waikato*, Hamilton 3240, New Zealand; 2 School of Psychology, University of New England, Armidale, NSW 2351, Australia

**Keywords:** time perception, temporal bisection, Weber’s law, dog, comparative cognition

## Abstract

**Simple Summary:**

Understanding the perceptual abilities of companion animals such as dogs adds to our understanding of the cognitive abilities of non-human animals. This study assessed the time perception abilities of dogs. In this study, dogs were required to identify whether the duration of a light was of a short or long duration by pressing a response lever. Dogs were able to correctly classify the durations as short or long. When given durations that were intermediate of the original short and long stimuli, their performance approached chance levels near the middle of the short and long durations. The performance of dogs on this task was similar to other animals, such as rats, pigeons and possums. Aspects of their performance also challenged some long-held assumptions of existing models of time perception. Research that assesses the cognitive abilities of dogs remains a fertile area of research that will improve our understanding about their abilities and limits.

**Abstract:**

Domestic dogs completed a temporal bisection procedure that required a response to one lever following a light stimulus of short duration and to another lever following a light stimulus of a longer duration. The short and long durations across the four conditions were (0.5–2.0 s, 1.0–4.0 s, 2.0–8.0 s, and 4.0–16.0 s). Durations that were intermediate, the training durations, and the training durations, were presented during generalization tests. The dogs bisected the intervals near the geometric mean of the short and long-stimulus pair. Weber fractions were not constant when plotted as a function of time: A U-shaped function described them. These results replicate the findings of previous research reporting points of subjective equality falling close to the geometric mean and also confirm recent reports of systematic departures from Weber’s law.

## 1. Introduction

Humans regularly utilize dogs for hunting, herding, general farm work, assisting police, aiding the disabled, and as companion animals [1]. Awareness of a dog’s ability to understand time could inform how people work with and train dogs. It could also test the generality of our understanding of psychological time from a comparative perspective as the majority of research on animal timing has primarily been based on work with traditional laboratory animals such as rats or pigeons. Recent reviews and commentaries on comparative psychology have noted the importance of the systematic evaluation of the cognitive abilities of a diverse range of species to add to our understanding of the similarities and differences across species as well as the appropriateness and utility of different methodologies (e.g., [2,3]).

Time perception is a fundamental cognitive ability and has been demonstrated in a variety of species, including rats (e.g., [4,5]), pigeons (e.g., [6,7,8]), fish (e.g., [9,10]), mice, (e.g., [11]) and possums [12]. The ability to time events and adapt to temporal change in one’s environment has considerable value to the survivability of an individual [13,14], and it has been argued that timing is critical to multiple aspects of sensation, perception, and behavior, and that different neurological structures are implicated in different aspects of timing [15]. However, the timing abilities of dogs remains a largely under-researched area, although there has been interest in their ability to perform a temporal bisection [16], the role of timing in decisions dogs make when foraging [17], and on timing and overshadowing [18].

Psychophysical procedures of various types have been used to study time perception by animals, such as the free-operant psychophysical choice procedure (e.g., [6,7]), and the pair-comparison method [8]. Temporal bisection tasks measure timing ability by requiring comparison of a stimulus duration against two reference durations that are typically the shortest and longest durations presented during training trials. Animals are usually trained to reach a specific level of accuracy before the addition of intermediate durations during testing (e.g., [5]).

Temporal bisection performance is typically described as a sigmoidal psychometric function when proportion of “long” responses are plotted as a function of stimulus duration. The duration that corresponds with 50% long responses is called the Point of Subjective Equality (PSE). The PSE represents the point between the two reference durations where the choice between the two alternatives is indifferent. The geometric mean of the two reference durations typically approximates the PSE (see [5,19,20]), and is a robust effect that has been shown to hold across multiple species, including pigeons [21,22], rats [5,23,24] and mice [21].

Weber’s law requires that the change in stimulus intensity that is necessary to deliver a constant level of discriminability is a constant proportion of the standard stimulus against which stimulus change is judged. As applied to temporal discrimination, this leads to predictions that timing accuracy is a relative rather than an absolute function of stimulus duration. Weber’s law has formed the cornerstone of theories of timing (e.g., [25,26]) and for which there is considerable empirical support (e.g., [27]). 

Relative accuracy is captured by dividing the just noticeable difference—the duration a stimulus needs to be changed by to be detected by the standard duration. If Weber’s law holds, then that ratio or fraction will be a constant and is commonly referred to as a Weber fraction (see [25,26,27,28,29]). In experiments where repeated estimates are made, the coefficient of variation (cv) is often taken to be equivalent to the Weber fraction (see [30]), where the cv is equal to the standard deviation of estimates divided by the mean of those estimates.

Failure of Weber’s law has been observed in several studies [30,31,32,33,34,35]. A generalized version of Weber’s law accounted for the failure to observe the predicted rise in the standard deviation for durations 2.0 s or longer [30]. Subsequently [31] demonstrated that Getty’s [30] Generalized Weber’s law did not account for their results, as the pigeons in their experiments produced Weber fractions that were U-shaped. Weber’s law and Generalized Weber’s law predict flat and J-shaped functions, respectively. 

Recently, Domeniconi and Machado [16] reported results from an exploratory study with dogs that were reinforced for correctly choosing a yellow stimulus after a short tone (1 s), or a choosing a blue stimulus after a long tone (4 s) on a temporal bisection task. They demonstrated that dogs were able to perform the task and produce timing performance when tested with durations intermediate the training stimuli. The dogs performed similarly to rats [5], monkeys [33], and other animals [12,26].

The present study employed a standard temporal bisection procedure and sought to test the timing ability of dogs across a range of duration pairs. Previous research would suggest that we should observe that the PSE will sit near the geometric mean of the short and long stimulus pairs. If Weber’s law holds, then Weber fractions should remain constant across the duration pairs, although a more reasonable prediction informed by the reports of others is that either a J- [30] or a U-shaped function [31] will describe the Weber fractions when plotted as a function of duration.

## 2. Materials and Methods 

### 2.1. Subjects

The subjects were six domestic dogs (three females and three males) of various mixed breeds. The dogs were all companion animals recruited from local dog owners known to the experimenters. The dogs were aged between 2 and 6 years old at the start of the experiment and were experimentally naïve. Water was available during each training session. The dog owners were asked not to feed their dogs on days before their participation in the experiment. The Animal Ethics Committee of the University of Waikato approved this research (AECN 855). 

### 2.2. Apparatus

A response box (575-mm long × 225-mm wide × 150-mm high) with three vertical metal response rods that protruded from the top of the box could record responses. The top panel of the response box was made of transparent Perspex, and the sides and floor were made of wood and painted white. The top panel was divided into three equally sized squares each with a vertical rod measuring (100-mm long × 10-mm in diameter). The left and right square could light up red or blue, and the whole top panel would light up green at the start of each trial. When activated a food dispenser (Manners Minder^®^, Knoxville, USA) dispensed 3–5 dog-food pellets. A customized program written in C++ controlled and recorded all experimental events through a PC laptop computer connected to the response box and the food dispenser.

### 2.3. Procedure

Training. Each dog received one session of magazine training during which dog-food pellets were delivered every 30 s for 30 min from the food dispenser. All the dogs ate reliably from the dispenser by the end of this training. 

In the next session, the dogs were trained to push the three vertical levers on the box using shaping by successive approximations. After a dog had made 10 consecutive reinforced responses to each lever it was then trained to press the middle lever to start each trial, which illuminated the panels green for 1.0 s, then the panel underneath, either the left or right panel, was illuminated blue, and reinforcer delivery followed the first lever press to the lever on the illuminated panel. When a dog correctly chose the blue illuminated lever on 70% of trials in a session, the training conditions were changed: The panels were then illuminated green for 4.0 s, then the panel underneath either the left or right panel was illuminated red, and reinforcer delivery followed the first level press to the lever on the illuminated panel. When the dog correctly chose the lever on the panel that was illuminated red on 70% of trials in a session, they then experienced five additional sessions where stimulus presentations alternated between 1 s and 4 s, and only the correct discriminative stimulus was subsequently presented. The correct discriminative stimulus was blue following a 1-s sample and red following a 4-s sample. The side of presentation of the discriminative stimuli on the response box was randomized across trials. 

#### 2.3.1. Discrimination Training

The dogs were trained to press the middle lever to begin each trial. Then the green sample stimulus was presented for either 1.0 s or 4.0 s. After the sample stimulus presentation, one side of the response panel was illuminated red, and the other side of the panel was illuminated in blue. The side of presentation of the red and blue stimuli on the response box was randomized across trials. After a response to one of the side levers, all panel lights were turned off. Responses to the middle lever during the choice phase of a trial had no scheduled effects. Correct responses resulted in reinforcer delivery, and incorrect responses did not. After a 1-s intertrial interval, the dog could then initiate the next trial by pressing the middle lever. An experimental session lasted for 50 trials. Two consecutive daily sessions of 80% correct or higher ended the discrimination training phase of the experiment.

#### 2.3.2. Testing

When dogs met the accuracy criteria for ending the training sessions, they experienced one test session for each of the four conditions. The other three conditions followed the same training and testing procedures. The duration pairs used during training were: 0.5 and 2.0 s, 2.0 and 8.0 s, and 4.0 and 16.0 s, respectively. In the testing phase, there were seven signal durations of the green light that were presented in each condition, the two durations used during training and five intermediate durations between those two extreme durations. The probability that an originally trained duration would be present was 0.25. Correct responses after the two original signal durations continued to be reinforced. Consequently, reinforcement was available on half the trials in a testing session, and the other half were unreinforced intermediate durations. 

## 3. Results

### 3.1. Proportion of Long Responses

**Figure 1:**
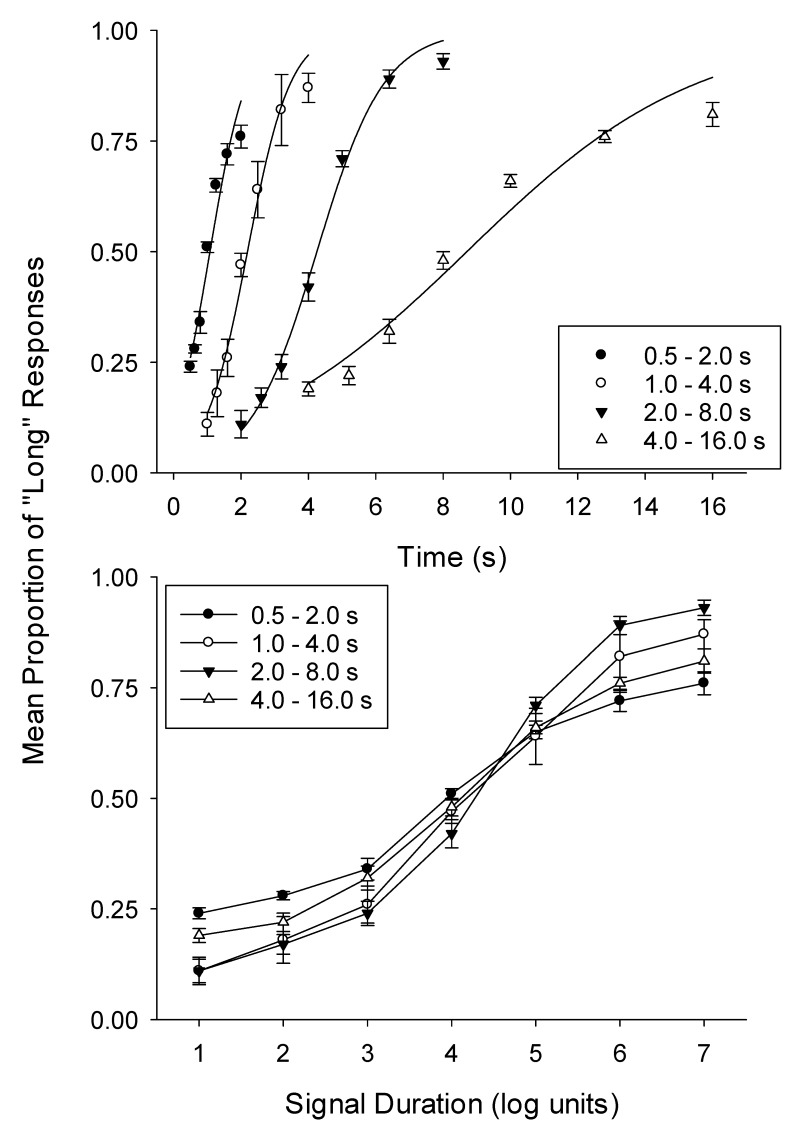
The top panel shows the proportion of “Long” responses plotted as a function of signal duration (s). The smooth lines through the data points were fit by Equation (1). The bottom panel shows the proportion of long responses as a function of time (top panel), as a function of signal duration in logarithmic units (bottom panel). The four duration ranges used were 0.5–2.0, 1.0–4.0, 2.0–8.0 and 4.0–16.0 s. The error bars are the standard error of the mean.

The proportion of long responses as a function of the sum of short and long responses was calculated at each duration in each condition for each dog. The proportion of long responses was lower for the shorter signal durations and increased as the signal duration increased for all dogs across all four conditions (see Figure 1). A Logistic Function (Equation (1)) was used to generate estimates of the mean (μ), and standard deviation (σ) of these psychometric functions for each dog in every condition.

(1) $$f={\left[1+\exp\left(\frac{\mu -t}{0.55\sigma }\right)\right]}^{-1}$$

The best fits of Equation (1) draw the smooth lines through the data points in the top panel of Figure 1 to the averaged proportion of long responses for each condition. Table 1 presents obtained parameter estimates derived from fitting Equation (1) to data from individual dogs for each condition using non-linear least squares regression. The fits of Equation (1) to the individual functions described the data well, except for two instances, the variance accounted for was always better than 0.900. However, Equation (1) tended to overestimate performance at the longer duration of each condition.

Figure 1 (bottom panel) shows the averages proportion of long responses plotted as a function of relative time: The functions do not superimpose. The 0.5–2.0-s condition had the flattest function, indicating that dogs found discriminating between the durations within this condition the hardest. The 2.0–8.0-s condition produced the steepest function, indicating that discriminability was high. Visual inspection of the bottom panel of Figure 1 confirmed that the PSE, where the probability of responding “Long” is 0.5 occurred at, or just past, the fourth signal duration, which corresponds to the geometric mean of the two trained durations in each condition.

### 3.2. Points of Subjective Equality

**Figure 2:**
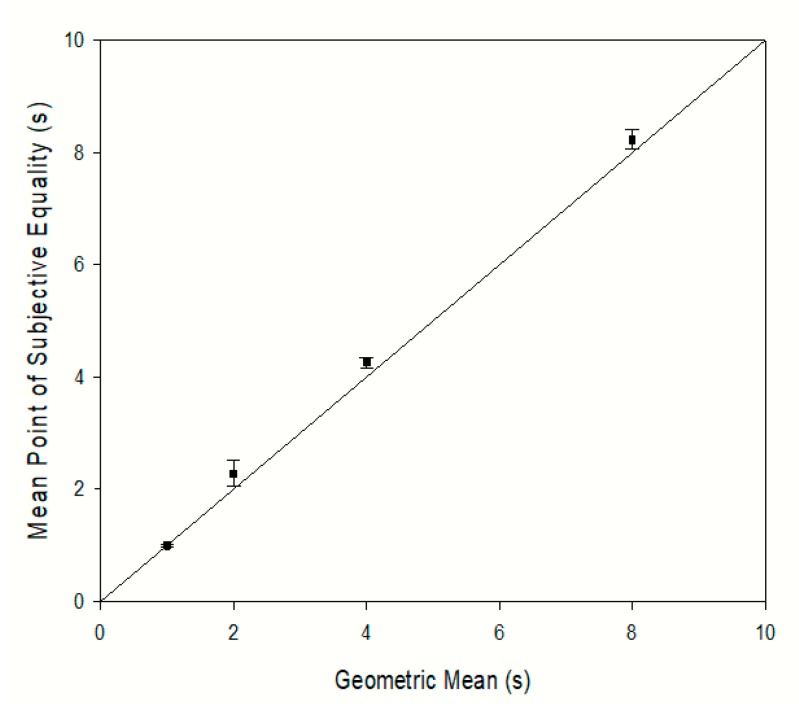
The Point of Subjective Equality (PSE) as a function of the geometric mean of the two trained extreme durations in each condition. The error bars are the standard error of the mean.

Figure 2 shows the PSE as a function of the geometric mean of the two extreme trained durations. PSEs were calculated by linear interpolation to provide the stimulus duration that would have corresponded with a 0.5 probability of responding “long” for each dog for each condition. The mean PSEs for each condition were 1.0 s (0.5–2.0 s), 2.28 s (1.0–4.0 s), 4.26 s (2.0–8.0 s) and 8.23 s (4.0–16.0 s), respectively. These values are close to the geometric means of 2.0 s, 4.0 s, and 8.0 s of the corresponding conditions.

### 3.3. Weber Fractions

**Figure 3:**
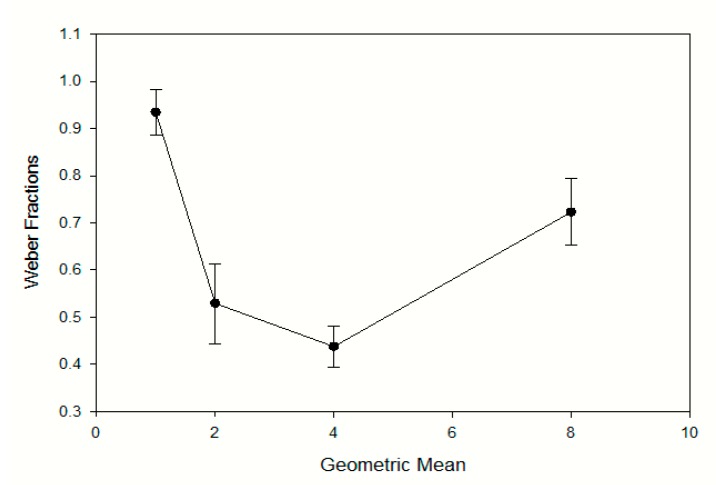
Weber fractions plotted as a function of the geometric mean across the four conditions. The error bars are the standard error of the mean.

Table 1 shows the parameter values from each dog under each condition, including the mean and standard deviation used to calculate the Weber fraction. The Weber fractions were calculated by dividing the standard deviation by the mean, were best described as a U-shaped function when plotted as a function of the geometric mean (see Figure 3) They were largest for the shortest condition, which was 0.5–2.0 s (0.935), smaller for the 1.0–4.0 s and 2.0–8.0 s conditions, respectively. They then increased again for the longest 4.0–16.0 s condition. Discrimination performance was best for durations in the range of 1.0–4.0 s and 2.0–8.0 s. 

## 4. Discussion

The results of the present study demonstrate new findings in the area of timing with dogs. Our study clearly shows their ability to discriminate between durations of different magnitudes. Across conditions, each subject had a lower proportion of “Long” responses at the shorter signal durations and progressed higher as the signal duration increased. The results of the present study are also consistent with those reported by [5]. However, in that paper [5], the rats were not tested on a signal duration of less than 1.0 s for comparison. This result of the present study are also consistent with those reported by [36] who found the accuracy of responding on their shortest signal range (0.5–1.0 s) significantly lower than their other three conditions (1.25–2.5 s, 4.0–8.0 s, 15.0–30.0 s). Further research could expand the range of durations, specifically the ability other dogs to time durations of less than a second, and also their sensitivity to durations in the order of tens of seconds and minutes, as that would provide insight into the mechanisms that control the timing of stimuli that differ by orders of magnitude.

The estimates of the PSE were closest to the geometric mean rather than the arithmetic or harmonic mean in all conditions, which replicates the findings of other temporal bisection procedures using rats [5,23,24], pigeons [22] and mice [21], where the geometric mean approximated the PSE. The use of dogs in this study, a species that has not been previously studied extensively in previous temporal bisection studies, supports the general conclusion that animals bisect stimulus durations pairs at their geometric mean.

In the present study, the ratio of the short to long training stimuli in for each condition was a constant 1:4 for all four conditions. The results show that as stimulus durations get longer, the Weber fractions get smaller and then increase again when the stimulus duration is very long. Figure 3 shows the Weber fractions as a function of the geometric means for the four conditions and is a U-shaped function. These findings are inconsistent with Weber’s law and its generalized form, which predict a flat function for Weber’s law or a reverse J-shaped function [30], respectively. However, they are consistent with reports from a growing number of research papers, such as [31,32], that have demonstrated the failure of Weber’s law to predict substantial increases in the standard deviation of longer duration ranges in a temporal bisection procedure. This study also adds support to the idea of a U-shaped function being a more accurate description of Weber fractions, as demonstrated by [31].

Developing a deeper understanding of the ability and extent that dogs can discriminate between stimuli of different durations has implications for training dogs in applied settings where the temporal context matters, such as where a dog needs to withhold a response or wait for a period of time before responding. Successful timing studies with dogs will enable faster and more accurate training in situations where timing is a critical part of a dog’s behavioral repertoire, such as responding with a specified time or having to withhold or hold a behavior for a fixed duration.

## 5. Conclusions

In conclusion, this study furthers our understanding of the timing abilities of dogs when the dogs were required to discriminate between durations in the order of fractions of a second to tens of seconds. The dogs showed a good ability to accurately discriminate between visual presentation of lights of different durations, and key aspects of their performance were consistent with published reports from other animals, such as rats [5] and pigeons [31], on similar tasks: The PSEs fell near the geometric mean of the duration pairs they were trained on (e.g., [5,19]); and Weber fraction were well described by a U-shaped function consistent with deviations from the scalar property timing reported by others (e.g., [31,32,37]).

Future research might explore the generality of dogs timing performance on other timing tasks that include temporal production, as well as categorization (e.g., [38]), and also systematically compare the performance of dogs with that of other species. The systematic assessment of the cognitive abilities of dogs remains a fertile area of research that will improve our understanding about their abilities and limits and also inform successful training procedures for the various roles dogs fulfil in society.

## Figures and Tables

**Table 1:** The mean, standard deviation (SD), Weber fractions (WF), R-Square (RSqr), and standard error of estimate (SEE) for all dogs, for each of the four conditions derived from nonlinear least squares regression fits of Equation (1) to data for individual dogs.

Condition	Dog	Mean	SD	WF	RSqr	SEE
0.5–2.0 s	1	1.065	0.875	0.821	0.980	0.037
	2	0.986	0.822	0.833	0.956	0.056
	3	1.261	1.295	1.027	0.890	0.067
	4	1.069	0.929	0.870	0.924	0.070
	5	1.078	1.004	0.932	0.934	0.062
	6	1.076	1.210	1.124	0.889	0.070
	Average	1.089	1.023	0.935	0.929	0.060
1.0–4.0 s	1	2.013	0.542	0.269	0.991	0.044
	2	2.043	0.789	0.386	0.979	0.058
	3	2.119	1.461	0.690	0.917	0.088
	4	2.222	0.838	0.377	0.983	0.053
	5	1.932	1.446	0.749	0.954	0.062
	6	3.110	2.188	0.704	0.904	0.070
	Average	2.240	1.211	0.529	0.955	0.063
2.0–8.0 s	1	4.355	1.383	0.317	0.999	0.016
	2	4.384	1.547	0.353	0.985	0.050
	3	3.827	2.260	0.591	0.979	0.047
	4	4.478	1.646	0.367	0.993	0.033
	5	4.050	1.897	0.468	0.994	0.029
	6	4.418	2.336	0.529	0.980	0.048
	Average	4.252	1.845	0.438	0.988	0.037
4.0–16.0 s	1	9.049	4.869	0.538	0.982	0.044
	2	8.617	4.730	0.549	0.973	0.054
	3	7.927	5.933	0.749	0.971	0.048
	4	8.919	6.433	0.721	0.961	0.055
	5	8.329	8.285	0.995	0.929	0.061
	6	9.249	7.658	0.828	0.916	0.073
	Average	8.682	6.318	0.730	0.955	0.054
